# Evaluation of Empirical and Machine Learning Approaches for Estimating Monthly Reference Evapotranspiration with Limited Meteorological Data in the Jialing River Basin, China

**DOI:** 10.3390/ijerph192013127

**Published:** 2022-10-12

**Authors:** Jia Luo, Xianming Dou, Mingguo Ma

**Affiliations:** 1 Chongqing Jinfo Mountain Karst Ecosystem National Observation and Research Station, School of Geographical Sciences, Southwest University, Chongqing 400715, China; 2 Chongqing Engineering Research Center for Remote Sensing Big Data Application, School of Geographical Sciences, Southwest University, Chongqing 400715, China

**Keywords:** reference evapotranspiration, empirical equations, complex extreme learning machine, relevance vector machine, extremely randomized trees, Jialing River Basin

## Abstract

The accurate estimation of reference evapotranspiration (*ET*_0_) is crucial for water resource management and crop water requirements. This study aims to develop an efficient and accurate model to estimate the monthly *ET*_0_ in the Jialing River Basin, China. For this purpose, a relevance vector machine, complex extreme learning machine (C-ELM), extremely randomized trees, and four empirical equations were developed. Monthly climatic data including mean air temperature, solar radiation, relative humidity, and wind speed from 1964 to 2014 were used as inputs for modeling. A total comparison was made between all constructed models using four statistical indicators, i.e., the coefficient of determination (*R*^2^), Nash efficiency coefficient (*NSE*), root mean square error (*RMSE*) and mean absolute error (*MAE*). The outcome of this study revealed that the Hargreaves equation (*R*^2^ = 0.982, *NSE* = 0.957, *RMSE* = 7.047 mm month^−1^, *MAE* = 5.946 mm month^−1^) had better performance than the other empirical equations. All machine learning models generally outperformed the studied empirical equations. The C-ELM model (*R*^2^ = 0.995, *NSE* = 0.995, *RMSE* = 2.517 mm month^−1^, *MAE* = 1.966 mm month^−1^) had the most accurate estimates among all generated models and can be recommended for monthly *ET*_0_ estimation in the Jialing River Basin, China.

## 1. Introduction

Reference crop evapotranspiration (*ET*_0_) is an essential element of the hydrological cycle. The accurate estimation of *ET*_0_ is critical for crop modeling, irrigation scheduling [1,2], and water resource management [3]. *ET*_0_ can be measured directly using lysimeters [4] and eddy covariance systems [5], which are expensive and time-consuming. As a more economical alternative to the direct measurement method, mathematical equations with measured meteorological parameters as inputs can be utilized to estimate *ET*_0_. The FAO-56 Penman–Monteith equation (FAO-56 PM) has been suggested by the Food and Agriculture Organization of the United Nations (FAO) as a standard model for estimating *ET*_0_ [6]. However, it is well known that using the FAO-56 PM equation requires many meteorological parameters, which limits its use in data-poor regions. In addition, the construction and maintenance of automatic meteorological stations is expensive, especially in developing countries [7]. Hence, simplified empirical equations with fewer input meteorological parameters are gaining popularity.

In recent decades, numerous researchers have developed various simplified empirical equations for estimating *ET*_0_. A detailed review of these empirical equations is beyond the scope of this study, and the several most widely used methods are pointed out as follows. The Hargreaves equation [8] was recommended as an alternative method for estimating *ET*_0_ in data-scarce regions [6]. The Hargreaves equation was also shown to be the most accurate model under warm humid and semi-arid climatic patterns [9]. The Truc equation [10] was determined to be the most appropriate model under cold humid and arid climates in Iran [11]. The Trabert [12], Romanenko [13], and Schendel [14] equations were reported to be the most promising equations for estimating *ET*_0_ under an arid climate in the Senegal River Valley [15]. The Irmak equation [16] can be successfully used to estimate *ET*_0_ in the humid climate in the Southeast United States. The Priestley–Taylor (PT) equation [17] is energy-driven and presented a good performance in estimating *ET*_0_. Numerous studies have developed these empirical equations in different climates and regions [18,19,20]. However, according to existing studies, the above-mentioned empirical methods may be limited due to the fact that the performance of empirical equations could significantly vary depending on the environment [21]. Therefore, in order to achieve reliable results, local calibration should be taken into account when applying the empirical equations above, and their modeling performance should be evaluated for obtaining the best model in the region.

*ET*_0_ is characterized by a complex nonlinear dynamic system and depends on various meteorological parameters and physical processes, so finding and establishing an accurate formula to illustrate all of those processes are challenging [22]. Fortunately, over the past few decades, machine learning (ML) algorithms as effective tools for dealing with nonlinear processes have already been successfully used in *ET*_0_ estimation. For instance, Citakoglu et al. [23] used the adaptive neuro fuzzy inference system (ANFIS), artificial neural network (ANN) models, and empirical methods including the Hargreaves and Ritchie [24] equations to estimate *ET*_0_ in Turkey. The results showed that the ANFIS model was the most reliable model. Feng et al. [25] compared an extreme learning machine (ELM), a generalized regression neural network (GRNN), and wavelet neural networks (WNNs) [26] versus empirical models (Hargreaves, Makkink [27], PT, Ritchie) in the humid area of Southwest China. The fundamental input meteorological parameter in these models was air temperature. The best results were obtained by ELM and GRNN with air temperature, sunlight duration, relative humidity, and wind speed as inputs. Fan et al. [28] studied the performance of support vector machine (SVM), ELM, random forest (RF), M5 model tree (M5Tree), extreme gradient boosting (XGBoost), and gradient boosting decision tree (GBDT) models for estimating *ET*_0_ in various climates of China. The results indicated that XGBoost and GBDT provided superior performance. Bellido-Jiménez et al. [29] evaluated multilayer perceptron (MLP), ELM, GRNN, SVM, RF, and XGBoost in southern Spain, with ELM as the most precise model. It should be noted that although the potential of the ML techniques mentioned above has been proven extensively for modeling *ET*_0_, these techniques still have various shortcomings, such as over-fitting for ANNs, and the high computational cost for SVMs. In conclusion, selecting an appropriate ML model for modeling *ET*_0_ is of essential importance.

In recent years, many attempts have been made to overcome the inherent drawbacks of traditional ML approaches in terms of their robustness, efficiency, and generalization performance. A number of new ML models have attracted attention in practical scientific issues, such as the relevance vector machine (RVM) [30], complex extreme learning machine (C-ELM) [31], and extremely randomized trees (ETRs) [32]. Deo et al. [33] compared ELMs, multivariate adaptive regression splines (MARS), and RVM to predict evaporative in Australia and concluded that the RVM model has a good ability compared to other traditional ML models. The RVM model has become an efficient tool in hydrology due to its excellent generalization properties [34]. Li et al. [31] inspected the performance of C-ELM and RBFNN in the application of channel equalization. Their study found that C-ELM had better results in the symbol error rate and learning speed. Saeed et al. [35] used SVM, RF, ANN, and ETR models to detect faults in wireless sensor networks. According to their study, the ETR model is robust against signal noise, with a strong reduction of bias and variance error. In addition, the ETR model has a shorter training time compared to other traditional ML models. To the best of our knowledge, however, the potential of these three relatively new ML methods has not been demonstrated for estimating *ET*_0_ in the humid area of Southwest China. Therefore, investigating and comparing the performance of RVM, C-ELM, and ETR approaches for *ET*_0_ modeling is a strong motivation for this study.

The present study therefore attempts to utilize three relatively new approaches and four empirical equations to estimate *ET*_0_ with limited meteorological data from 1964 to 2014 in the Jialing River Basin, China. More specifically, the aims of this study are the following: (1) to investigate the practicability and ability of RVM, C-ELM, and ETR models for estimating monthly *ET*_0_ at seven meteorological stations; (2) to test the validity of four empirical equations (Hargreaves, Schendel, Irmak, and Romanenko) for estimating monthly *ET*_0_ in the Jialing River Basin, China; (3) to evaluate the relative importance of meteorological variables for *ET*_0_ estimates by the use of various combinations and determine the best combination as input for ML models; (4) to compare the predictive ability of our developed ML models with four empirical equations using four statistical indicators.

## 2. Materials and Methods

### 2.1. Study Region and Data Collection

**Figure 1:**
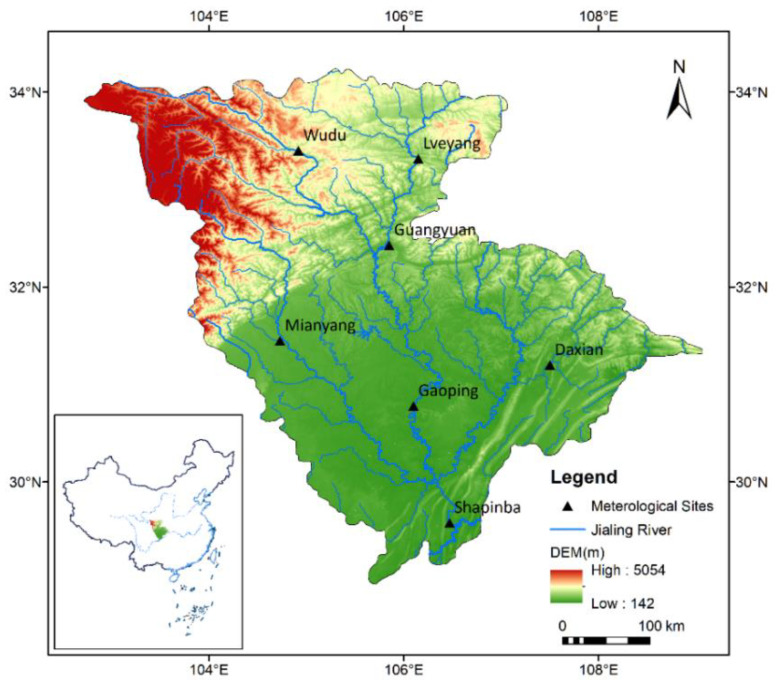
The geographical location of the study area.

The Jialing River Basin (JRB) (29°17′ N–34°28′ N and 102°35′ E–109°01′ E) originates from the northern side of the Qinling Mountains and covers an area of 160,000 km^2^. The Jialing River has a total length of nearly 1120 km, flowing through four provinces: Shanxi, Gansu, Sichuan, and Chongqing. Figure 1 shows the geographical location of the JRB with meteorological stations. JRB has a subtropical humid monsoon climate, and the average annual rainfall is between 900 and 1200 mm [36]. The mean air temperature is around 25.5 °C during the summer months from June to August and falls to 6.5 °C during the winter months from December to February [37].

**Figure 2:**
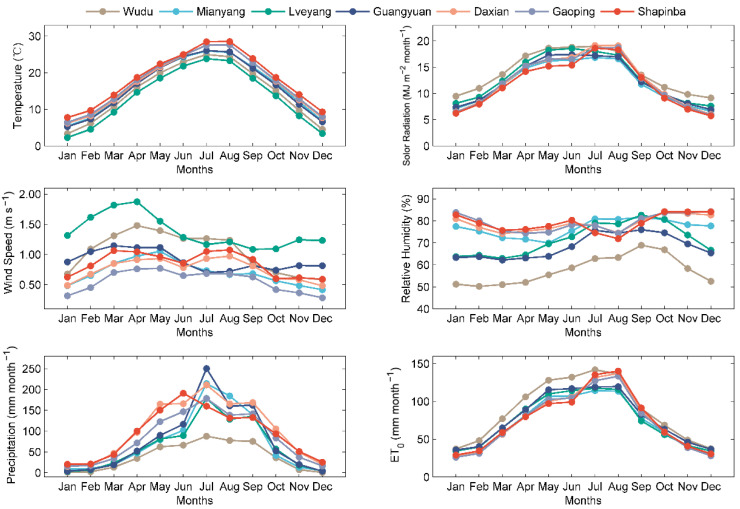
Monthly variations of meteorological parameters and *ET*_0_ for seven studied meteorological stations during 1964–2014.

The present study was conducted using seven stations in the JRB: Wudu, Mianyang, Lveyang, Guangyuan, Daxian, Gaoping, and Shapinba. The period from 1964 to 2014 was selected due to the availability of climatic data with few gaps. Monthly climatic data were used in this investigation to estimate monthly *ET*_0_. The monthly *ET*_0_ values have obvious periodicity [38] and play an important role in planning long-term irrigation management [39]. Therefore, estimating monthly *ET*_0_ using machine learning methods is necessary and feasible. The geographical coordinates and the monthly average meteorological parameters are reported in Table 1. Figure 2 shows the monthly variation of the mean air temperature ($T_{mean}$), solar radiation ($R_{s}$), wind speed at 2 m height ($u_{2}$), relative humidity (RH), precipitation, and standard FAO-56 PM *ET*_0_ of these meteorological stations. These meteorological datasets were provided by the China Meteorological Data Service Centre (see http://data.cma.cn/en, accessed on 14 April 2021).

### 2.2. Penman–Monteith Method

The FAO-recommended Penman–Monteith equation (FAO-56 PM) [6] is employed to estimate *ET*_0_ data. Given the absence of lysimeter-measured *ET*_0_ data, the FAO-56 PM equation is an accepted and widely used practice [40,41]. Thus, the FAO-56 PM equation is considered the benchmark model for the calibration and evaluation of the Hargreaves, Schendel, Irmak, Romanenko, C-ELM, RVM, and ETR models. The equation is given below [6]:

(1) $$ET_{0}=\frac{0.408\Delta \left(R_{n}-G\right)+\gamma \frac{900}{T_{mean}+273}u_{2}\left(e_{s}-e_{a}\right)}{\Delta +\gamma \left(1+0.34u_{2}\right)}$$

where $ET_{0}$ is the standardized grass reference evapotranspiration (mm month^−1^), Δ is the slope of the vapor pressure curve (kPa °C^−1^), $R_{n}$ is the net radiation at the crop surface (MJ m^−2^ month^−1^), G is the soil heat flux (MJ m^−2^ month^−1^), $T_{mean}$ is the mean air temperature (°C), $u_{2}$ is the wind speed at 2 m height (m s^−1^), $e_{s}$ is the saturation vapor pressure (kPa), $e_{a}$ is the actual vapor pressure (kPa), γ is the air psychometric constant (kPa °C^−1^).

### 2.3. Empirical Equations

Four empirical equations were employed to estimate monthly *ET*_0_*:* Hargreaves, Schendel, Irmak, and Romanenko. These equations were locally calibrated using the FAO-56 PM equation to optimize their performance.

The Hargreaves equation [9] is one of the easiest and most accurate equations for estimating *ET*_0_. It is described as

(2) $$ET_{0}=a_{1}\cdot R_{s}\cdot (T_{mean}+a_{2})$$

where $ET_{0}$ is the reference evapotranspiration (mm month^−1^), $a_{1}$ and $a_{2}$ are the empirical coefficients, $R_{s}$ is the solar radiation (MJ m^−2^ month^−1^), $T_{mean}$ is the monthly mean air temperatures (°C).

The Schendel equation, as described by Schendel [14] is as follows:

(3) $$ET_{0}=\frac{a_{1}\cdot T_{mean}}{RH}$$

where $ET_{0}$ is the reference evapotranspiration (mm month^−1^), $a_{1}$ is the empirical coefficient, $T_{mean}$ is the monthly mean air temperature (°C), RH is the relative humidity (%).

Irmak equation [16] is a linear regression equation and can be expressed as

(4) $$ET_{0}=a_{1}\cdot R_{s}+a_{2}\cdot T_{mean}-a_{3}$$

where $ET_{0}$ is the reference evapotranspiration (mm month^−1^), $a_{1}$, $a_{2}$ and $a_{3}$ are the empirical coefficients, $R_{s}$ is the solar radiation (MJ m^−2^ month^−1^), $T_{mean}$ is the monthly mean air temperature (°C).

The Romanenko equation proposed by Romanenko [13] is used to estimate *ET*_0_ based on the mean air temperature and relative humidity. It is expressed as

(5) $$ET_{0}=a_{1}\cdot {\left(a_{2}+T_{mean}\right)}^{a_{3}}\cdot (a_{4}-RH)$$

where $ET_{0}$ is the reference evapotranspiration (mm month^−1^), $a_{1}$, $a_{2}$, $a_{3}$, and $a_{4}$ are empirical coefficients, $T_{mean}$ is the monthly mean air temperature (°C), RH is the relative humidity (%).

### 2.4. Relevance Vector Machine

Tipping [30] proposed the relevance vector machine (RVM) as a general-purpose sparse Bayesian modeling method. The RVM model applies automatic relevance determination (ARD) to linear regression to remove parameters that contribute nothing to the construction of the model, resulting in a sparsity model. Therefore, models generated by the RVM are usually more concise than those generated by the corresponding SVM, increasing the speed of processing test data. In the RVM model, most parameters converge to zero during the iterative learning process, while non-zero parameters correspond to points referred to as relevance vectors, which reflect the most essential features of the dataset. The RVM model can utilize non-Mercer kernels and has a fast computation speed compared to the SVM model. Moreover, the RVM model has a high generalization capacity and generates probabilistic interpretation and prediction uncertainties [42]. The kernel function of the RVM model used in this study is sigmoid.

### 2.5. Complex Extreme Learning Machine

The Complex Extreme Learning Machine (C-ELM) is an ELM-based model developed by Li et al. [31], which has better generalization capability than ELM. The C-ELM model is simple to use and offers a faster learning rate, faster reaction time, and a low symbolic error rate (SER) [43]. When constructing a C-ELM model, the hidden layer bias and input weights (linking the input and hidden layers) are produced at random, and the final output weights (connecting the hidden and output layers) are simply determined mathematically rather than being iteratively tuned. This method eliminates the potential for human error in manually fine-tuning control parameters including starting weights, learning rates, and learning epochs. The analysis of excellent solutions using the C-ELM model can also help prevent local minima. The hyperparameter tested in this study is the hidden layers. The remaining parameters are in the default settings.

### 2.6. Extremely Randomized Trees

Random Forest (RF) uses a randomized with a put-back approach to obtain the training set of each decision tree, which results in duplicate samples in the training set. The RF model does not guarantee that all samples can be fully utilized, and there may be similarities among decision trees [44]. Based on the above considerations, the extreme random tree (ETR) model was developed by Geurts [32]. Every decision tree in the ETR model is trained utilizing the entire training set, guaranteeing that the training set is fully used and minimizing the final prediction bias. To ensure structural differences among decision trees, the division threshold of each feature is randomly chosen from the sub-datasets, and the feature with the best division is chosen as the satisfactory division attribute by the specified threshold. Therefore, the ETR model is trained faster than the RF. The hyperparameters tested in this study are the number of trees, the number of points for each leaf, and the number of attributes selected to perform the random splits. The rest of the parameters are in the default mode.

### 2.7. Model Development

In the present study, the RVM, C-ELM, and ETR models were developed and compared with four calibrated empirical equations. *ET*_0_ is only affected by climatic data including $T_{mean}$, $R_{s}$, RH, $u_{2}$, etc. [45]. Temperature and solar radiation data have been proved to be important factors for *ET*_0_ estimation [46]. This is consistent with the background theory that temperature and solar radiation are the two main driving forces of *ET*_0_ [47]. In the development of ML models, it is desirable to determine the independence of each parameter to reduce complexity and increase efficiency [48]. Utilizing the Pearson’s correlation coefficient is one of the commonly used approaches to check the independence of hydrological parameters. This is a parametric technique, and therefore its application requires the conditions that must be respected in the data set. One of the most crucial assumptions is that the statistical distribution is normal, which is not always true in hydrological data [49]. Additionally, the presence of outlier data may have an impact on the modeling outcomes. As a result, instead of utilizing Pearson’s correlation coefficient to check the independence of the input parameters, this study estimated the accuracy of the ML models with six input combinations of parameters ($T_{mean}$, $R_{s}$, RH, and $u_{2}$) based on the background theory to assess the significance of each climatic parameter and train the ML models.

Table 2 illustrates the six input combinations used in this study. The RVM3, C-ELM3, ETR3, Hargreaves, and Schendel equations have the same input combination ($T_{mean}$, $R_{s}$). The RVM4, C-ELM4, ETR4, Irmak, and Romanenko equations have the same input combination ($T_{mean}$, RH).

The entire dataset (data during 1964–2014) was divided into three parts. The first part (data during 1964–2000) was used to train/calibrate the ML models/empirical equations; the second part (data during 2001–2007) was utilized to validate the ML models and empirical equations; the third part (data during 2008–2014) was used to test the calibrated ML models and revised empirical equations. This approach can ensure a high generalization ability and an independent test of the calibrated models [50,51]. Although it is disappointing that the dataset from 2015 to present was not used, similar studies in Iran [49,52] illustrated that 50 years of data are sufficient to meet the objectives of the study.

### 2.8. Performance Evaluation

This study employed four frequently used statistical metrics to compare empirical equations and ML models to the FAO-56 PM equation: correlation of determination (*R*^2^), Nash–Sutcliffe efficiency coefficient (*NSE*), root mean square error (*RMSE*), and mean absolute error (*MAE*). They are defined as

(6) $$R^{2}=\frac{(\sum_{i=1}^{n}(ET_{0,PM,i}-\bar{ET_{0,PM,i}})(ET_{0,e,i}-\bar{ET_{0,e,i}}){)}^{2}}{\sum_{i=1}^{n}{\left(ET_{0,PM,i}-\bar{ET_{0,PM,i}}\right)}^{2}\sum_{i=1}^{n}{\left(ET_{0,e,i}-\bar{ET_{0,e,i}}\right)}^{2}}$$

(7) $$NSE=1-\frac{\sum_{i=1}^{n}{\left(ET_{0,PM,i}-ET_{0,e,i}\right)}^{2}}{\sum_{i=1}^{n}{\left(ET_{0,PM,i}-\bar{ET_{0,PM,i}}\right)}^{2}}$$

(8) $$RMSE=\sqrt{\frac{1}{n}\sum_{i=1}^{n}{\left(ET_{0,PM,i}-ET_{0,e,i}\right)}^{2}}$$

(9) $$MAE=\frac{1}{n}\sum_{i=1}^{n}\left|ET_{0,PM,i}-ET_{0,e,i}\right|$$

where n is the sample number, $ET_{0,PM,i}$ is the standard FAO-56 PM *ET*_0_ value, $ET_{0,e,i}$ is the model estimated *ET*_0_ value, $\bar{ET_{0,PM,i}}$ is the average FAO-56 PM *ET*_0_ value, and $\bar{ET_{0,e,i}}$ is the average model estimated *ET*_0_ value.

## 3. Results and Discussion

### 3.1. Estimation of Empirical Models

Table 3 represents the statistical indices of the four empirical equations averaged over the seven stations. In the testing period, the Hargreaves equation is superior to other empirical equations; the *R*^2^, *NSE*, *RMSE*, and *MAE* values are calculated as 0.982, 0.957, 7.047 mm month^−1^, and 5.946 mm month^−1^, respectively, followed by the Irmak equation (*R*^2^ = 0.973, *NSE* = 0.953, *RMSE* = 7.588 mm month^−1^, *MAE* = 5.983 mm month^−1^), and the Romanenko equation in third place (*R*^2^ = 0.966, *NSE* = 0.916, *RMSE* = 9.648 mm month^−1^, *MAE* = 7.612 mm month^−1^). The Schendel equation has the poorest results (*R*^2^ = 0.944, *NSE* = 0.901, *RMSE* = 11.025 mm month^−1^, *MAE* = 8.900 mm month^−1^).

**Figure 3:**
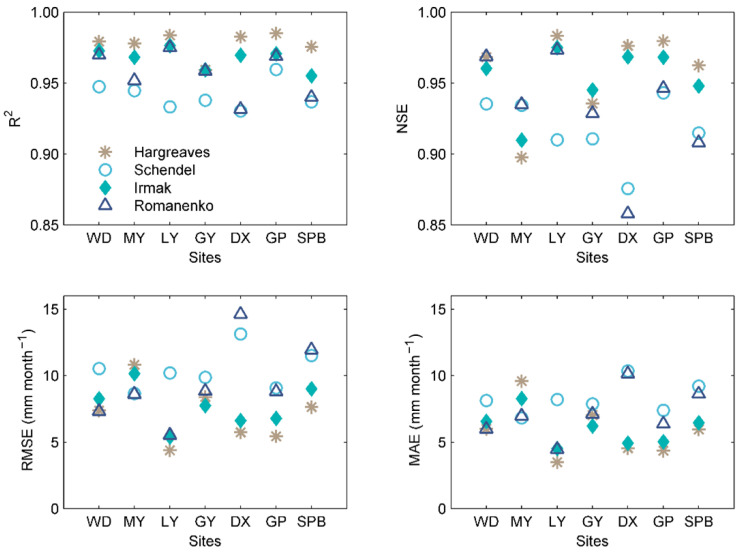
Statistical indices of the four calibrated empirical equations at the seven stations for modeling *ET*_0_ during the testing period (2008–2014). WD-Wudu, MY-Mianyang, LY-Lveyang, GY-Guangyuan, DX-Daxian, GP-Gaoping, SPB-Shapinba.

The *R*^2^, *NSE*, *RMSE*, and *MAE* values of the studied empirical equations at different stations are given in Figure 3. In general, the Hargreaves equation performs best at all stations, followed by the Irmak equation. The Romanenko equation is slightly better than the Schendel equation at most stations. The lowest *NSE* value and highest *RMSE* value are found at the Daxian station, which has higher precipitation (1228.38 mm) than those of other stations (most < 1000 mm). With the lowest *RMSE* and *MAE* values and the highest *R*^2^ and *NSE* values, Lveyang station achieves the best performance.

**Figure 4:**
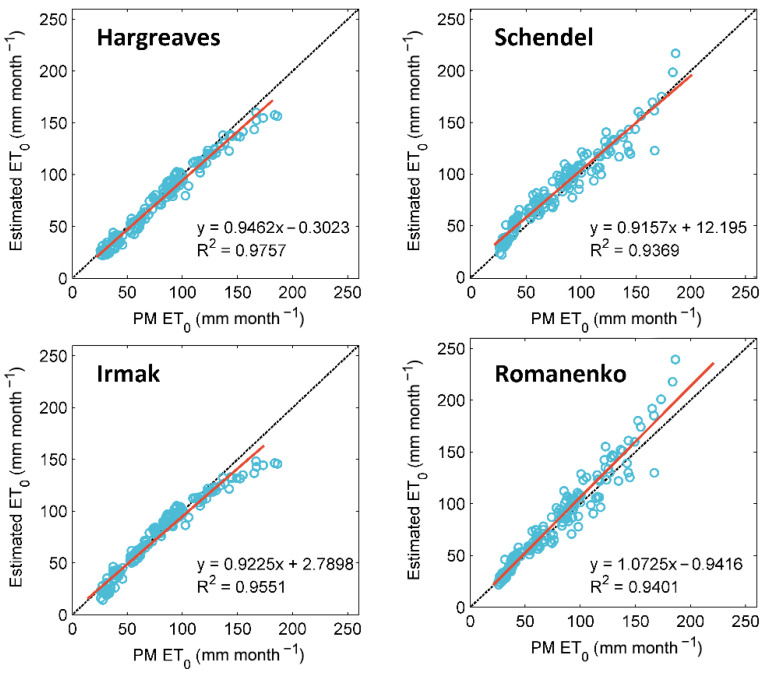
Scatter plots of *ET*_0_ values estimated by the four empirical models against FAO-56 PM *ET*_0_ values at Shapinba station during the testing period.

The Shapinba station is a typical station belonging not only to the Jialing River basin but also to the Three Gorges Reservoir area of the Yangtze River. Figure 4 shows the FAO-56 PM *ET*_0_ values and empirical equation-estimated *ET*_0_ values at Shapinba station during the testing period. The scatter plot of the Hargreaves equation provides accurate results between 0 and 150 mm month^−1^ but underestimates *ET*_0_ when values exceed 150 mm month^−1^. The Irmak equation performs well from 50 to 150 mm month^−1^ and underestimates *ET*_0_ when values are less than 50 mm month^−1^ and greater than 150 mm month^−1^. The Romanenko equation significantly overestimates *ET*_0_ and shows more scattered estimates than the others. The Schendel equation overestimates low *ET*_0_ values and underestimates high *ET*_0_ values. Therefore, the Hargreaves equation is the most accurate of the four empirical models. Our results are consistent with those of Moeletsi et al. [53] and Valipour et al. [54].

### 3.2. Estimation of Machine Learning Models

Table 4 is created to compare the accuracy of *ET*_0_ estimated by the RVM, C-ELM, and ETR models with six input combinations. From Table 4, it is seen that input combination 6 has superior performance to other input combinations. Considering all eighteen ML models, the best model is C-ELM6 (*R*^2^ = 0. 995, *NSE* = 0. 995, *RMSE* = 2.517 mm month^−1^, *MAE* = 1.966 mm month^−1^), the best model with three input parameters is C-ELM5 (*R*^2^ = 0.974, *NSE* = 0.943, *RMSE* = 8.293 mm month^−1^, *MAE* = 6.570 mm month^−1^), the best model with two input parameters is C-ELM3 (*R*^2^ = 0.985, *NSE* = 0.966, *RMSE* = 6.153 mm month^−1^, *NSE* = 4.988 mm month^−1^), and the best model with only one input parameter is RVM2 (*R*^2^ = 0.946, *NSE* = 0.909, *RMSE* = 10.626 mm month^−1^, *MAE* = 8.652 mm month^−1^).

**Figure 5:**
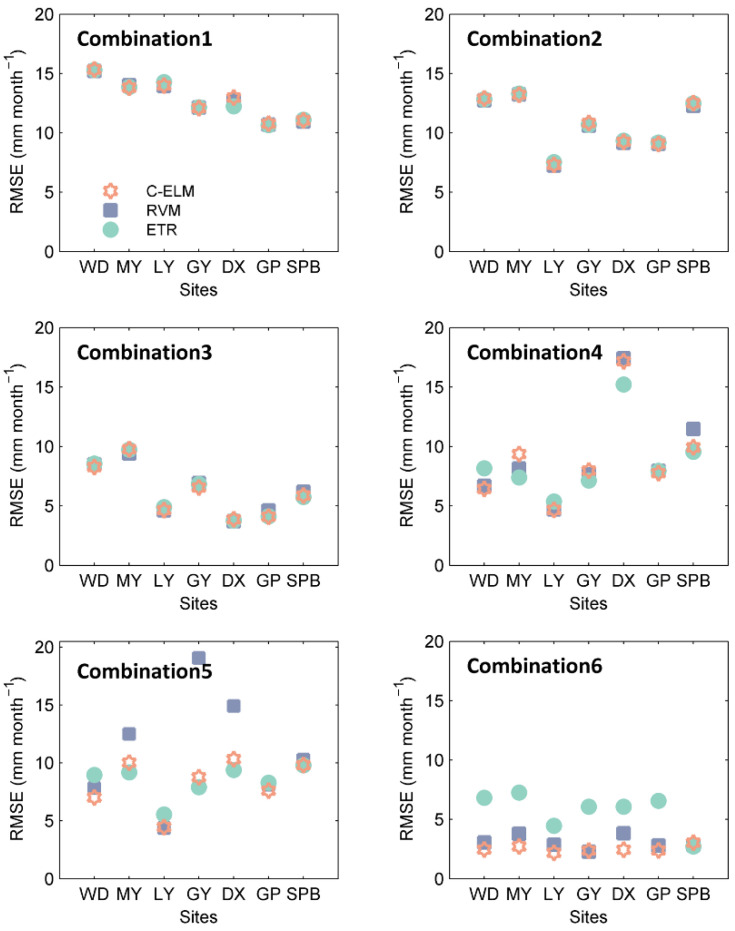
*RMSE* values of the RVM, C-ELM, and ETR models with six input combinations at the seven stations for modeling *ET*_0_ during the testing period (2008–2014). WD-Wudu, MY-Mianyang, LY-Lveyang, GY-Guangyuan, DX-Daxian, GP-Gaoping, SPB-Shapinba.

Figure 5 shows the *RMSE* values of the RVM, C-ELM, and ETR models with six input combinations. According to Figure 5, when input parameters are combinations 1–4, the RVM, C-ELM, and ETR models present similar estimation accuracy. When input parameters are combinations 5–6, the C-ELM model gives better performance than the RVM and ETR models. Otherwise, combination 2 gives lower *RMSE* values than those of combination 1. The combination 3 also gives quite good results. The *RMSE* of combination 5 has little significant improvement compared to combination 4. The results show that $R_{s}$ has the greatest effect on estimating monthly *ET*_0_ in this humid study area, followed by $T_{mean}$ and RH. The minimum effective input parameter is determined as $u_{2}$ for the RVM, CELM, and ETR models.

**Figure 6:**
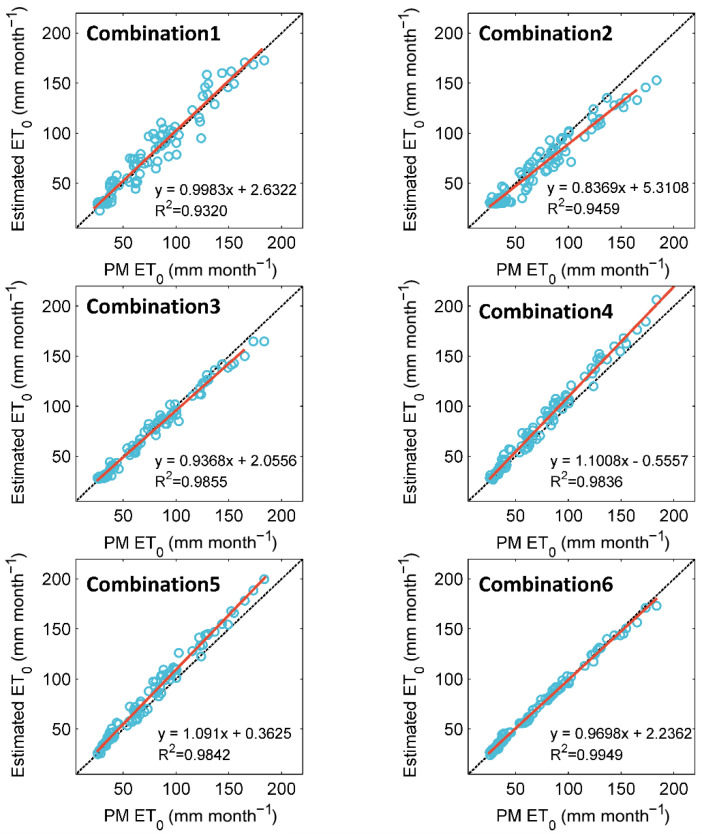
Scatter plots of *ET*_0_ values estimated by the C-ELM model against FAO-56 PM *ET*_0_ values at Shapinba station during the testing period.

From Table 4 and Figure 5, the C-ELM model is superior to the RVM and ETR models. Figure 6 is intended to investigate the accuracy of the C-ELM model with six input combinations. As can be seen from Figure 6, the scatter plot of C-ELM6 is less scattered. Among the models investigated, the C-ELM6 model has the best fit line and the highest *R*^2^ value (*R*^2^ = 0.9949). The C-ELM4 and C-ELM5 models invariably overestimate the *ET*_0_ values. The *R*^2^ values of the C-ELM4 and C-ELM5 are 0.9836 and 0.9842, respectively. The fitting slopes of C-ELM1, C-ELM2, and C-ELM3 are between 0.83 and 0.99, indicating those models underestimate the *ET*_0_ values. The C-ELM1 and C-ELM2 models with one input parameter are more scattered than the other models. The C-ELM3 model with two input parameters is less distributed and produces reliable results (*R*^2^ = 0.9855). Overall, C-ELM3 and C-ELM6 are recommended to estimate monthly *ET*_0_ values.

### 3.3. Comparison of Empirical and Machine Learning Models

**Figure 7:**
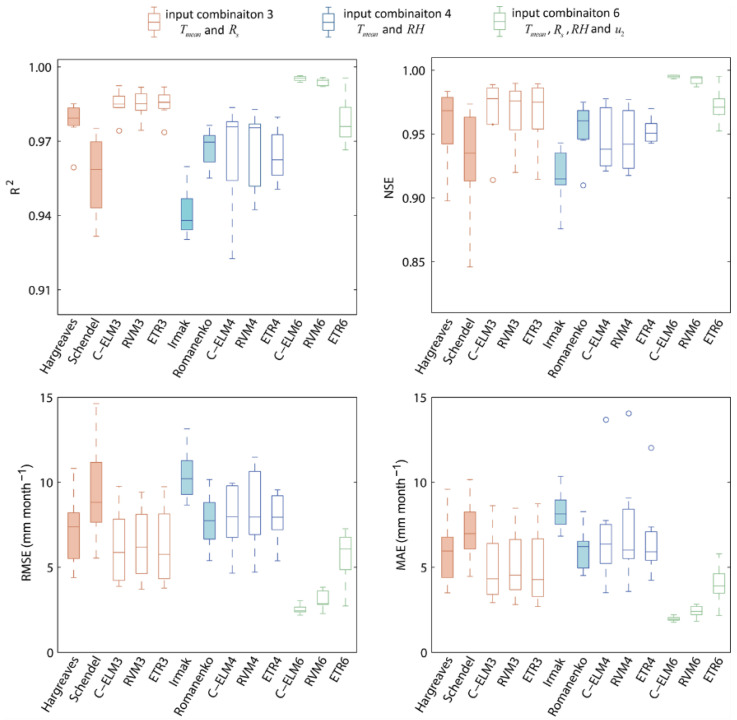
Boxplot of statistical indices for the four empirical equations and ML models for predicting *ET*_0_ with input combinations 3, 4, and 6. Solid boxes are empirical equations, and blank boxes are ML models.

Figure 7 presents the boxplots of statistical indices for the four empirical equations and studied ML models with input combinations 3, 4, and 6. As shown in Figure 7, the C-ELM6, RVM6, and ETR6 models offer the best outcomes compared to other empirical equations and ML models. The C-ELM6 model has the highest values for *R*^2^ and *NSE* and the lowest values for *RMSE* and *MAE*, followed by the RVM6 model, with the ETR6 model in third place. When $T_{mean}$ and $R_{s}$ are the inputs, The C-ELM3, RVM3, and ETR3 models give better performance than the Hargreaves and Schendel equations. When $T_{mean}$ and RH are the inputs, the C-ELM3, RVM3, ETR3, and Romanenko equations have similar accuracy, but the Irmak equation has the worst performance. In conclusion, the ML models exhibit superior performance compared to the four empirical equations, and the C-ELM6 model is the best model.

**Figure 8:**
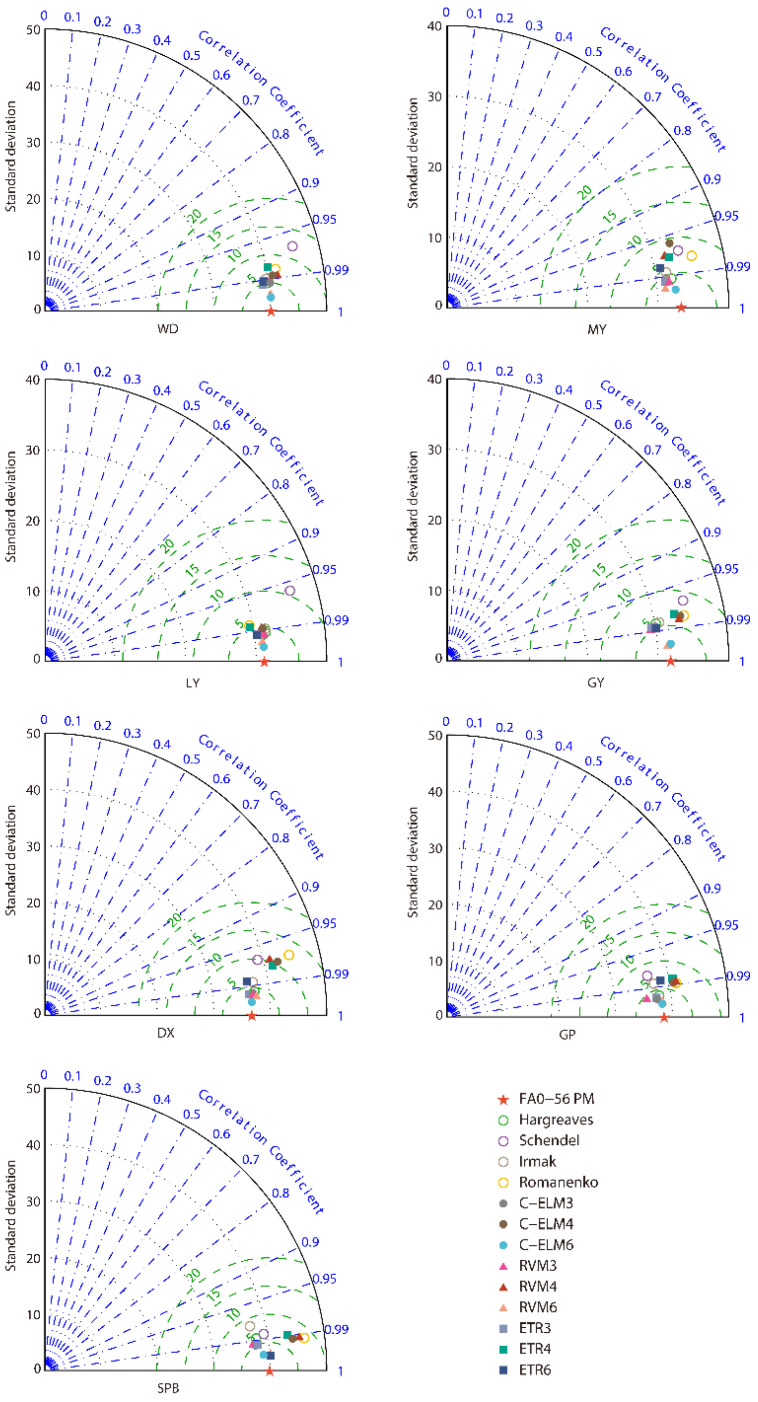
Taylor diagrams for the four empirical equations and RVM, C-ELM, and ETR models for predicting *ET*_0_ at the seven stations.

The Taylor diagram is created to study the adaptation between FAO-56 PM *ET*_0_ and estimated *ET*_0_ (Figure 8). As displayed in Figure 8, the C-ELM6, RVM6, and ETR6 models perform quite well. The most accurate model compared to FAO-56 PM *ET*_0_ for the seven stations except Shapinba is the C-ELM6 model. At the Shapinba station, the best model is the ETR6 model. Overall, the C-ELM6 model and Schendel equation yield the highest and lowest accordance, respectively, with the FAO-56 PM standard deviation line.

## 4. Conclusions

In this study, for the first time, four empirical equations (Hargreaves, Schendel, Irmak, and Romanenko) and three ML models (RVM, C-ELM, and ETR) were established for estimating monthly *ET*_0_ in the JRB. Monthly meteorological datasets including $T_{mean}$, $R_{s}$, RH, and $u_{2}$ from the seven meteorological stations in this Basin for 1964–2014 were used. Based on the obtained results, some conclusions can be drawn.

Firstly, $R_{s}$ and $T_{mean}$ were considered to have significant effects on *ET*_0_, especially under humid conditions. RH and $u_{2}$ produced less meaningful results when used separately. Nevertheless, adding RH and $u_{2}$ to $R_{s}$ or $T_{mean}$ improved the accuracy for estimating monthly *ET*_0_.

Secondly, four empirical equations (Hargreaves, Schendel, Irmak, and Romanenko) had acceptable estimation accuracy. The Hargreaves equation was the best empirical equation in the JRB. However, all ML models had more accurate results compared to empirical equations. Therefore, it can be confirmed that the RVM, C-ELM, and ETR models are efficient methods to get satisfactory results for estimating monthly *ET*_0_. The RVM and ETR models showed similar ability. The C-ELM performed better than the RVM and ETR models. Moreover, the C-ELM6 model, which uses $T_{mean}$, $R_{s}$, RH, and $u_{2}$ as inputs, produced the best estimates.

Finally, this study conclusively substantiated the effectiveness and generalization performance of ML techniques (RVM, C-ELM, and ETR) for modeling and forecasting *ET*_0_, which was also the first attempt to investigate the JRB with a subtropical humid monsoon climate. Nevertheless, under other climatic conditions, the applicability and validity of these advanced regression techniques remain to be investigated. More importantly, with the development of remote sensing techniques, using satellite remote sensing datasets combined with meteorological datasets needs to be studied to further improve the accuracy of ML models.

## Figures and Tables

**Table 1:** Meteorological stations with monthly average climatic conditions (from 1964 to 2014). $T_{mean}$ is the monthly mean air temperature (°C), $R_{s}$ is the monthly solar radiation (MJ m^−2^ month-1), $u_{2}$ is the monthly wind speed at 2 m height (m s^−1^), RH is the monthly relative humidity (%), *ET*_0_ is the monthly standard FAO-56 PM *ET*_0_ values (mm month-1), and precipitation is the average annual rainfall (mm).

Sites	Latitude (° N)	Longitude (° E)	Altitude (m)	$T_{mean}$	$R_{s}$	$u_{2}$	RH	*ET*_0_	Precipitation
Wudu	33.40	104.92	1079.1	14.82	14.12	1.54	57.65	87.66	466.29
Mianyang	31.45	104.73	522.7	16.45	11.96	1.20	76.87	70.95	865.49
Lveyang	33.32	106.15	794.2	13.50	12.99	1.87	71.64	74.42	776.57
Guangyuan	32.43	105.85	513.8	16.26	12.40	1.40	68.38	77.47	939.32
Daxian	31.20	107.50	344.3	17.24	12.47	1.25	78.80	73.99	1228.38
Gaoping	30.78	106.10	309.7	17.48	12.32	1.06	79.34	72.79	1005.38
Shapinba	29.58	106.47	259.1	18.40	11.83	1.35	79.16	74.71	1121.27

**Table 2:** Summary of the six input combinations of the meteorological parameters used in the RVM, C-ELM, and ETR models.

Combination	RVM	C-ELM	ETR	Input Combination
1	RVM1	C-ELM1	ETR1	$T_{mean}$
2	RVM2	C-ELM2	ETR2	$R_{s}$
3	RVM3	C-ELM3	ETR3	$T_{mean}$ $,\;R_{s}$
4	RVM4	C-ELM4	ETR4	$T_{mean}$ , RH
5	RVM5	C-ELM5	ETR5	$T_{mean}$ , RH $,\;u_{2}$
6	RVM6	C-ELM6	ETR6	$T_{mean}$ $,\;R_{s}$ , RH $,\;u_{2}$

**Table 3:** Statistical indices of the studied empirical equations for modeling *ET*_0_ at the seven stations.

Model		Hargreaves	Schendel	Irmak	Romanenko
Calibration	*R* ^2^	0.98	0.942	0.973	0.964
	*NSE*	0.979	0.929	0.973	0.964
	*RMSE*	5.201	9.689	6.027	6.959
	*MAE*	3.983	7.766	4.581	5.381
Validation	*R* ^2^	0.976	0.946	0.965	0.956
	*NSE*	0.958	0.932	0.954	0.941
	*RMSE*	7.079	9.621	7.77	8.751
	*MAE*	5.763	7.679	6.012	6.587
Testing	*R* ^2^	0.982	0.944	0.973	0.966
	*NSE*	0.957	0.901	0.953	0.916
	*RMSE*	7.047	11.025	7.588	9.648
	*MAE*	5.946	8.9	5.983	7.612

**Table 4:** Statistical indices for the RVM, C-ELM, and ETR models with six input combinations for modeling *ET*_0_ during the training, validation, and testing periods.

	Model	RVM1	RVM2	RVM3	RVM4	RVM5	RVM6	C-ELM1	C-ELM2	C-ELM3	C-ELM4	C-ELM5	C-ELM6	ETR1	ETR2	ETR3	ETR4	ETR5	ETR6
Training	*R* ^2^	0.912	0.943	0.986	0.972	0.976	0.993	0.913	0.944	0.986	0.972	0.978	0.994	0.922	0.948	0.988	0.976	0.981	0.993
	*NSE*	0.912	0.943	0.985	0.972	0.948	0.993	0.913	0.944	0.986	0.972	0.978	0.994	0.922	0.948	0.988	0.976	0.981	0.993
	*RMSE*	10.740	8.748	4.313	6.153	7.409	2.977	10.682	8.693	4.195	6.099	5.362	2.709	10.113	8.423	3.948	5.661	5.063	2.955
	*MAE*	8.460	6.927	3.190	4.550	5.797	2.120	8.418	6.869	3.076	4.499	3.867	1.908	7.941	6.662	2.834	4.244	3.736	2.063
Validation	*R* ^2^	0.890	0.935	0.979	0.957	0.952	0.992	0.886	0.935	0.981	0.959	0.968	0.994	0.883	0.934	0.981	0.957	0.950	0.972
	*NSE*	0.886	0.908	0.967	0.950	0.908	0.991	0.882	0.906	0.969	0.949	0.951	0.993	0.879	0.907	0.968	0.953	0.941	0.967
	*RMSE*	12.414	11.020	6.318	8.100	9.956	3.406	12.665	11.081	5.975	8.177	7.906	2.978	12.826	11.095	6.189	7.935	8.610	5.996
	*MAE*	9.890	8.845	4.912	6.066	7.819	2.571	10.032	8.893	4.719	6.082	6.026	2.268	10.073	8.830	4.838	6.012	6.520	4.314
Testing	*R* ^2^	0.890	0.946	0.985	0.967	0.969	0.994	0.890	0.945	0.985	0.965	0.974	0.995	0.890	0.944	0.985	0.965	0.962	0.978
	*NSE*	0.866	0.909	0.965	0.926	0.881	0.992	0.865	0.907	0.966	0.927	0.943	0.995	0.866	0.907	0.965	0.937	0.944	0.972
	*RMSE*	12.815	10.626	6.287	9.148	11.016	3.069	12.860	10.720	6.153	9.034	8.293	2.517	12.800	10.773	6.246	8.688	8.446	5.716
	*MAE*	10.001	8.652	5.106	7.208	9.077	2.396	10.049	8.742	4.988	6.993	6.570	1.966	9.966	8.715	5.040	6.668	6.465	4.000

## Data Availability

All data required to evaluate the conclusions in the research are present in the paper. Additional data related to this research may be requested from the authors.
